# On Efficient Deployment of Wireless Sensors for Coverage and Connectivity in Constrained 3D Space †

**DOI:** 10.3390/s17102304

**Published:** 2017-10-10

**Authors:** Chase Q. Wu, Li Wang

**Affiliations:** 1 Department of Computer Science, New Jersey Institute of Technology, Newark, NJ 07102, USA; 2 College of Engineering, Xi’an International University, Xi’an 710077, Shaanxi, China; wangli@xaiu.edu.cn

**Keywords:** sensor deployment, network connectivity, *k*-coverage, approximation algorithm

## Abstract

Sensor networks have been used in a rapidly increasing number of applications in many fields. This work generalizes a sensor deployment problem to place a minimum set of wireless sensors at candidate locations in constrained 3D space to *k*-cover a given set of target objects. By exhausting the combinations of discreteness/continuousness constraints on either sensor locations or target objects, we formulate four classes of sensor deployment problems in 3D space: deploy sensors at Discrete/Continuous Locations (D/CL) to cover Discrete/Continuous Targets (D/CT). We begin with the design of an approximate algorithm for DLDT and then reduce DLCT, CLDT, and CLCT to DLDT by discretizing continuous sensor locations or target objects into a set of divisions without sacrificing sensing precision. Furthermore, we consider a connected version of each problem where the deployed sensors must form a connected network, and design an approximation algorithm to minimize the number of deployed sensors with connectivity guarantee. For performance comparison, we design and implement an optimal solution and a genetic algorithm (GA)-based approach. Extensive simulation results show that the proposed deployment algorithms consistently outperform the GA-based heuristic and achieve a close-to-optimal performance in small-scale problem instances and a significantly superior overall performance than the theoretical upper bound.

## 1. Introduction

Sensor networks have been widely used in many agricultural, military, and industrial applications. The technological advances in both sensing and communication have significantly improved the quality of sensors at a reduced cost, making it possible to deploy more sensors than before to achieve quality through quantity. The redundancy in the deployment of sensors is often explored to cover targets of interest with multiple sensors for fault tolerance and sustained operation.

Depending on the nature of the environment and the type of the application, sensors could be deployed in either a deterministic or a stochastic manner. In the former, sensors are typically mounted manually at some predetermined locations to meet a certain deployment objective; while in the latter, sensors are not bound to any specific location and are oftentimes dropped randomly by vehicles or airplanes to cover a large geographical region. In the past decade, a significant number of research efforts have been made in both of the deployment scenarios for various purposes, e.g., to localize potential targets in the former [1] and maximize sensing coverage with network connectivity in the latter [2].

In this paper, we consider a specific type of wireless sensor networks (WSNs) that are deployed in challenged environments for microclimate monitoring, such as cultural heritage sites with historical frescos, sculpture paintings, or religionary statues, which are typically located on the 3D surface of constrained space [3,4]. One important requirement on sensor deployment in such environments is to provide a full coverage of target objects with a high level of sensing reliability for the purpose of assuring their long-term conservation. Similar sensor network applications could be also found in structured indoor environments such as a display cabinet or exhibition hall in a museum. This requirement is commonly defined as the *k*-coverage problem, where the degree of coverage is *k* such that every point in a target region is covered with at least *k* sensors.

This paper generalizes and investigates a sensor deployment problem in constrained 3D space to achieve *k*-coverage for a given set of target objects with a minimal number of sensors whose deployments are limited to some specific (candidate) locations. Note that the coverage problem on a 2D plane is a special case of constrained 3D space. By exhausting the combinations of discreteness/continuousness constraints on either candidate sensor locations or target objects in 3D space, we formulate four classes of problems on sensor deployment: (i) discrete candidate sensor locations to cover discrete target objects (DLDT); (ii) discrete candidate sensor locations to cover continuous target objects (DLCT); (iii) continuous candidate sensor locations to cover discrete target objects (CLDT); and (iv) continuous candidate sensor locations to cover continuous target objects (CLCT). We conduct an in-depth investigation into the computational complexity of these problems and prove all of them to be NP-complete. We first design a greedy strategy-based approach to DLDT and provide its approximation ratio of (lnn+1), where *n* is the number of target points, and then convert the other three problems to DLDT through discretization. For DLCT, we first discretize the continuous target objects into a set of divisions, which are treated as discrete points without sacrificing sensing precision, and then adapt the approximation algorithm for DLDT to DLCT. We provide a rigorous proof that the number of divisions intersected by *m* circles in a 2D plane is tightly upper bounded by $\left(m^{2}-m+1\right)$, and then prove that the algorithm for DLCT has an approximation ratio of (ln(k·n·m)+1), where *m* denotes the number of candidate locations for possible sensor deployment. For CLDT, we discretize the continuous candidate sensor locations and design an approximate algorithm in a similar way with the same approximation ratio as DLDT. Finally, for CLCT, we take a hexagon-based discretization approach to discretize the continuous candidate sensor locations or target objects into a number of hexagons, and then covert this problem to either DLCT or CLDT.

For each of these four problems, we further consider a connected version where all sensors in the deployment region must be connected for communication, referred to as C-DLDT, C-DLCT, C-CLDT, and C-CLCT. We first study a subproblem, i.e., Steiner Tree Problem with Minimum number of Steiner Points on Constrained Locations (STP-MSPCL), and design an approximate algorithm based on minimum spanning tree with an approximation ratio of 5 in a 2D plane and 12 in 3D space. We solve C-DLDT using an approximate algorithm with an approximation ratio of (lnn+13), which integrates the greedy approach for DLDT and the approximate algorithm for STP-MSPCL. We design algorithms for C-DLCT and C-CLDT, and derive their approximation ratios in a similar way.

We implement the proposed deployment algorithms and evaluate their performance in a simulated setting. For performance comparison, we also design and implement an optimal solution based on an integer linear programming (ILP) formulation, which is only meant for small-scale problem instances, and a genetic algorithm (GA)-based approach. Extensive simulations show that these algorithms consistently outperform the GA-based heuristic and achieve a close-to-optimal performance in small-scale problem instances and a significantly superior overall performance than the theoretical upper bound. These results shed light on the efficiency of the proposed deployment schemes and their great potential for practical sensor network applications. For example, many cultural relics in the world such as the ancient Great Wall and Terracotta Army in China are suffering from serious environmental pollution and degradation. Deploying a network of sensors in the constrained surroundings to measure environmental parameters such as humidity, temperature, and lighting is a key component of a viable solution for protection from environment-related threats. The proposed algorithms could be used to produce cost-effective sensor deployment schemes in such real-life scenarios.

Compared to the conference version [5], we have made substantial improvements to our work and new contributions to the field as follows:

- We developed a method based on integer linear programming (ILP) to find the lower bound of the optimal solution to the DLDT problem by formulating it as an ILP task.
- We derived a tighter approximation ratio of Algorithm 1 for DLDT based on the lower bound $OPT_{LP}$ of OPT by solving the LP problem in advance.
- We provided a detailed rigorous proof on the upper bound on the number of divisions intersected by *m* circles in a 2D plane.
- We derived a more accurate approximation ratio of the algorithm designed for DLCT with target object discretization.
- We designed a completely new approach to CLCT, which first divides the CLCT problem into two subproblems, and then converts the two subproblems to CLDT and DLCT, respectively.
- We implemented the proposed algorithms and evaluated the performance against the optimal solution and a genetic algorithm-based approach through extensive simulations.

The rest of the paper is organized as follows. Section 2 describes related work. Section 3 defines the problems on constrained sensor deployment in 3D space. The approximate algorithms with and without the connectivity requirement are proposed in Section 4 and Section 5, respectively. We evaluate the performance of the proposed algorithms in Section 6 and conclude our work in Section 7.

## 2. Related Work

Sensor deployment for coverage and connectivity in 2D space has been investigated in-depth in the literature. Many efficient techniques have been proposed, including several recent efforts based on integer linear programming (ILP) models and artificial intelligence such as randomization-based genetic algorithm [6,7]

as well as bee colony algorithm and particle swarm optimization [8]. We focus our survey on the techniques that are directly related to our work, specifically targeting 3D space and considering both coverage and connectivity.

The traditional 3D *k*-coverage problem is tackled by filling up the space with multiple non-overlapping copies of an already *k*-covered 3D shape or model. Along this line of research, Ammari proposed the Reuleaux tetrahedron model to characterize the 3D *k*-coverage problem and a sensor deployment strategy to achieve full *k*-coverage of a 3D field [9]. These results were also published in [10] where network connectivity is considered. More recently, Pal and Medhi designed a new shape termed as Sixsoid to tessellate a 3D field of interest [11]. Our work is somewhat opposite to theirs in that we attempt to *k*-cover the 3D surfaces of target objects in constrained space. Unaldi  et al. proposed a deployment strategy for 3D terrains based on wavelet transformation, where sensors are relocated by the mutation operator of the genetic algorithms (GAs) [12]. Similar to many other heuristics, such as artificial intelligence techniques do not ensure a performance bound.

There also exist a number of efforts on sensor deployment for *k*-coverage in 3D space where the deployment of sensors is limited to some specific candidate locations or the entire region of interest. In [13], Wang  et al. conducted a study on cost minimization of sensor placement in a bounded 3D region *R* comprised of discrete points. They consider several sensor types with different sensing ranges and costs, and attempt to find a subset of points to place a selection of sensors to cover every point in region *R* with at least *k* sensors with a minimal total cost. In [14], Andersen  et al. studied the problem of deploying identical sensors in a region *R* that constitutes continuous 3D space. Their approach is to discretized *R* into grid points that are covered by sensors to be placed. Apparently, such a discretization process would result in the loss of precision for continuous region coverage, which does not guarantee *k*-coverage of the entire region *R*. In [15], Huang  et al. proposed an algorithm to determine whether every point in the target area is covered by at least *k* sensors under the assumption that sensors are already deployed. In [16], Zhao  et al. proposed a surface coverage model where the target region is a complex 3D surface and sensors can be only deployed at some discrete points on the surface.

In [17], Funke  et al. proposed to select a set of deployed sensors of minimum cardinality to achieve sensing coverage and maintain network connectivity. They designed a greedy approach, which has an approximation ratio no better than O(logn), where *n* is the number of sensors. They also proposed a solution to provide an approximate coverage in a specific case where the number of selected sensors is limited by a constant factor far from the optimal value. Steiner tree algorithm has been used in various contexts to connect deployed sensors that have already achieved the required coverage of the region. Gupta  et al. investigated the minimal connected cover set (MCCS) problem [18], which has been shown to be NP-complete, where a minimal subset of connected nodes fully cover the entire region. The time complexity of their solution is resolution dependent as they approximated the sensor coverage area with a set of square units. Zhou  et al. investigated the connected *k*-coverage problem in [19] and designed a distributed greedy approach. Note that they treated the target object or query region in the same manner as in [18], and did not discuss the computational complexity.

The problems we study in this paper differ from the aforementioned research efforts in several aspects: (i) We consider a set of constrained sensor deployment problems in 3D space; (ii) We exhaust the combinations of various deployment constraints posed on candidate sensor locations and target objects, which can be either discrete points or continuous areas; (iii) We require the deployed sensors to *k*-cover a given set of target objects and also maintain communication connectivity. We design a set of efficient algorithms to discretize continuous candidate sensor locations or target objects without loss of sensing precision. More importantly, we propose an approximation algorithm with a bounded performance ratio for each of these problems, which have been proved to be NP-complete.

## 3. Problem Formulation

Our network model considers sensors with a certain sensing radius $r_{s}$ and communication radius $r_{c}$. The following definitions are provided on sensor coverage.

**Definition** **1.** **cover**: a sensor s (or a sensor located at point l) covers a target object t if and only if the Euclidean distance between s (or l) and any point on t is less than or equal to $r_{s}$.

**Definition** **2.** **k-cover**: A set S of sensors (or a set L of sensor locations) k-cover a target object t if there are at least k sensors in S (or at least k sensor locations in L) that cover t.

We study the constrained sensor deployment problem in 3D space, which is formulated as an optimization problem: Given a 3D convex region *R* (e.g., a cave on a historical relic site or an exhibition room in a museum) with certain spatial constraints for sensor deployment and a set *T* of *n* separated target objects within region *R*, our goal is to deploy a minimum number of sensors at the candidate locations on the *inner surface* of *R* to achieve *k*-coverage for each target object. Due to the spatial constraints, sensors can be only placed at a finite set *L* of *m* separated locations. According to the type of location *L* and target *T*, we define four constrained sensor deployment problems in 3D space as follows:

- Discrete *L* with Discrete *T* (DLDT), i.e., both the feasible sensor locations and the present target objects are discrete points.
- Discrete *L* with Continuous *T* (DLCT), i.e., the feasible sensor locations are discrete points and the 3D surfaces of the present target objects are composited by a finite set of continuous convex 2D areas.
- Continuous *L* with Discrete *T* (CLDT), i.e., the feasible sensor locations fall on a finite set of continuous convex 2D areas in 3D space and the target objects are discrete points.
- Continuous *L* with Continuous *T* (CLCT), i.e., both the feasible sensor locations and the surfaces of the present target objects are a finite set of continuous convex 2D areas in 3D space.

Note that in the above problems, the convex 2D areas for continuous target objects and feasible sensor locations could be positioned anywhere in 3D space (i.e., they may not be located on the same plane). At each discrete point for sensor deployment, we can deploy at most one sensor. When a feasible sensor location is a continuous 2D area, we can deploy a sensor at any position within that area. Furthermore, we consider a connected version of each of the four sensor deployment problems where all deployed sensors must be connected, referred to as C-DLDT, C-DLCT, C-CLDT, and C-CLCT.

## 4. Algorithm Design without Connectivity Requirement

We first prove the NP-completeness of DLDT and propose an approximate algorithm with an approximation ratio of (lnn+1) as a base solution. We use a discretization approach to covert DLCT, CLDT, and CLCT to DLDT.

### 4.1. Algorithm Design for DLDT

We show that the known NP-complete Discrete Unit Disk Cover (DUDC) problem [20] is a special case of DLDT. DUDC is a well-studied problem and is defined as follows: Given a set *P* of points in a 2D plane, and a set *D* of unit disks at some fixed locations, the goal is to find a minimum-cardinality subset $D^{\prime }\subseteq D$ to cover all points of *P*. Note that DUDC is essentially a geometric version of the set cover problem, where the sets are defined as a collection of unit disks.

**Theorem** **1.** The DLDT problem is NP-complete.

**Proof.**  We can restrict DLDT to DUDC by allowing only those instances where k=1, $r_{s}=1$, and *L* and *T* are in the same 2D plane. The proof by restriction for NP-completeness is established in [21], where the “restriction” should be imposed on the inputs (i.e., problem space), not the question. Since a special case (DUDC) of DLDT is NP-complete, so is DLDT. ☐

We develop a method based on integer linear programming (ILP) to find the lower bound of the optimal solution to the DLDT problem by formulating it as an ILP task. Let variable $x_{l}$ represent the decision of whether or not to deploy a sensor at a feasible sensor location l∈L: $x_{l}=1$ if a sensor is deployed at location *l*; $x_{l}=0$, otherwise. For each target point t∈T, let S(t) denote the set of candidate sensor locations in *L* that can cover target point *t*. The ILP task for the DLDT problem is formulated as follows:

(1) $$Objective:\mathrm{Min}(\sum_{l\in L}x_{l}),$$

subject to

(2) $$\forall t\in T,\sum_{l\in S(t)}x_{l}\geq k,$$

(3) $$\forall l\in L,x_{l}\in \left\{0,1\right\}.$$

In the above ILP task, the objective is to minimize the total number of sensors to be deployed. The first constraint ensures that each target point is *k*-covered, and the second constraint requires that each $x_{l}$ variable to be either 0 or 1. Since the ILP problem is NP-hard, this formulation does not help solve the sensor deployment problem under study. However, the ILP formulation leads to a way of finding the lower bound of the optimal solution through the LP-relaxation technique. We relax the integer constraint of Equation (3) in the ILP formulation to obtain an LP formulation:

(4) $$\forall l\in L,0\leq x_{l}\leq 1.$$

Let OPT and $OPT_{LP}$ be the optimal objective values of the ILP and LP problems, respectively. We have $OPT_{LP}\leq OPT$, since the solution space for the LP problem is a superset of that for the ILP problem. Therefore, $OPT_{LP}$ provides a lower bound to the optimal solution to the DLDT problem. According to the *k*-coverage constraint in Equation (2), we have OPT≥k and $OPT_{LP}\geq k$.

Andersen  et al. modified the greedy algorithm originally designed for the Set Cover problem [14] to solve the sensor deployment problem with multiplicity *k*, but no approximation ratio was provided. We adopt a similar greedy strategy to DLDT, referred to as GreedyDLDT, as shown in Algorithm 1, whose output is a set $L_{0}$ of candidate locations selected for sensor deployment. We use function $U(l,T,L_{0})$ to compute a set of target points in *T*, which are covered by *l* but not *k*-covered by $L_{0}$. GreedyDLDT follows an iterative procedure to select a candidate sensor location covering the largest possible number of target points that are not *k*-covered at each stage, until all target points in *T* are *k*-covered. GreedyDLDT has a time complexity of $O(m^{2}\cdot n$) in the worst case. When k=1, it is the same as the approximate algorithm designed for the Set Cover problem, and Johnson already proved that the approximation ratio of this algorithm is upper bounded by (lnn+1) [22]. When k≥1, we shall prove that the approximation ratio of GreedyDLDT is also upper bounded by (lnn+1), as stated in Theorem 2.

**Algorithm 1** GreedyDLDT Input: *L*, *T*, *k* Output: A set $L_{0}$ of selected sensor locations1:$L_{0}=\emptyset$;2:**while** $L_{0}$ does not *k*-cover *T* **do**3: $l^{*}=\underset{l\in L-L_{0}}{\mathrm{argmax}}(|U\left(l,T,L_{0}\right)\left|\right)$;4: $L_{0}=\left\{l^{*}\right\}\cup L_{0}$;5:**return** $L_{0}$;

**Theorem** **2.** The approximation ratio of Algorithm 1 is (lnn+1).

**Proof.**  Considering that each of *n* target points is covered by at least *k* sensors, there should be in total k·n target points returned by $U(l^{*},T,L_{0})$ in GreedyDLDT in Algorithm 1, where each target point has a multiplicity of *k*. We use $T^{\prime }=\left\{t_{1}^{\prime },t_{2}^{\prime },\ldots ,t_{k\cdot n}^{\prime }\right\}$ to denote the set of target points that are numbered in the order they are covered, and use $T_{i}^{\prime }=T^{\prime }-CAT_{j=1}^{j=i}U\left(l_{j}^{*},T,L_{0}\right)$, where CAT is defined as an operation to concatenate multiple sets into a single one without removing duplicate elements, to denote the set of target points that have not yet been covered after the *i*-th iteration, with $T_{0}^{\prime }=T^{\prime }$.

We use OPT to denote the minimum number of sensor locations selected to *k*-cover all target points. When OPT=1, it is obvious that *k* must be equal to 1 because there is only one selected location for sensor deployment. There must exist a sensor location $l^{*}$ that can cover all *n* target points, and hence this greedy procedure identifies $l^{*}$ in the first iteration and achieves the optimality in this case. We consider OPT≥2 in the rest of our proof.

After the *i*-th iteration, we know that the remaining target points $T_{i}^{\prime }$ can be covered by at most OPT sensor locations, and each location should cover $\frac{|T_{i}^{\prime }|}{OPT}$ target points on average. The principle of Pigeon Hole indicates that there must exist one sensor location $l\in (L-L_{0})$, which can cover at least $\left\lceil \frac{|T_{i}^{\prime }|}{OPT}\right\rceil$ target points; otherwise, the OPT sensor locations cannot *k*-cover $T_{i}^{\prime }$. In this greedy method, we always select the sensor location $l_{i+1}^{*}$ that covers the largest number of target points in $T_{i}^{\prime }$. Therefore, $|T_{i}^{\prime }\left|-\right|T_{i+1}^{\prime }|\geq \left\lceil \frac{|T_{i}^{\prime }|}{OPT}\right\rceil \geq \frac{|T_{i}^{\prime }|}{OPT}$, or $|T_{i+1}^{\prime }|\leq \left(1-\frac{1}{OPT}\right)\left|T_{i}^{\prime }\right|$. Starting from $|T_{0}^{\prime }|=k\cdot n$, an inductive procedure leads to $|T_{i}^{\prime }|$ as follows:

(5) $$|T_{i}^{\prime }|\leq {\left(1-\frac{1}{OPT}\right)}^{i}\cdot k\cdot n.$$

We use *q* to denote the number of iterations this approach takes to satisfy the following equation:

(6) $${\left(1-\frac{1}{OPT}\right)}^{q}\cdot k\cdot n=OPT.$$

By rearranging ${\left(1-\frac{1}{OPT}\right)}^{q}$ and OPT, and taking natural logarithm on both sides of Equation (6), we obtain:

(7) $$\ln\left(\frac{k\cdot n}{OPT}\right)=q\cdot \ln\left(1+\frac{1}{OPT-1}\right).$$

We consider a function $f\left(x\right)=\ln\left(1+\frac{1}{x-1}\right)-\frac{1}{x}$. Since f(2)>0, $\lim_{x\to +\infty }f\left(x\right)=0$, and its first-order derivative $f^{\prime }\left(x\right)=\frac{-1}{x^{2}\cdot \left(x-1\right)}<0$, we know that f(x) is a monotonic decreasing function when x≥2. It follows that f(x)≥0 or $\ln\left(1+\frac{1}{x-1}\right)\geq \frac{1}{x}$ when x≥2, i.e., $\ln\left(1+\frac{1}{OPT-1}\right)\geq \frac{1}{OPT}$ when OPT≥2. Therefore, based on Equation (7), we obtain q≤OPT·ln(k·n/OPT).

In Algorithm 1, after *q* iterations, $|T_{q}^{\prime }|\leq {\left(1-\frac{1}{OPT}\right)}^{q}\cdot k\cdot n=OPT$, which means that only OPT target points in $T^{\prime }$ have not yet been covered. Since each iteration covers at least one target point in $T^{\prime }$, we can fully cover $T^{\prime }$ after $\left\lceil q\right\rceil +OPT$ iterations, i.e., all *n* target points are *k*-covered. Algorithm 1 terminates after $\left\lceil q\right\rceil +OPT$ iterations, and produce the number $|L_{0}|$ of selected sensor locations as:

(8) $$\begin{array}{cc} |L_{0}|\leq & \left\lceil q\right\rceil +OPT<OPT\cdot \left(\ln\frac{k\cdot n}{OPT}+1\right)\\ \leq & OPT\cdot (\ln n+1). \end{array}$$

The inequality in Equation (8) holds because OPT≥k, as shown by the constraint in Equation (2) in the LP problem. Hence, the approximation ratio of GreedyDLDT in Algorithm 1 is upper bounded by (lnn+1). ☐

We would like to point out that DLDT with k=1 is exactly the Set Cover problem, which has been proven to be inapproximable in polynomial time within a factor of O(lnn), unless P=NP [23]. Accordingly, O(lnn) is the best approximation ratio achievable for DLDT when k≥1. However, we are able to produce a tighter approximation ratio in Theorem 3 if we compute the lower bound $OPT_{LP}$ of OPT by solving the LP problem in advance.

**Theorem** **3.** Algorithm 1 has an approximation ratio of $\left(\ln\frac{k\cdot n}{OPT_{LP}}+1\right)$, if the lower bound $OPT_{LP}$ of OPT is pre-computed.

**Proof.**  Since $OPT_{LP}\leq OPT$, from Equation (8), we have

(9) $$|L_{0}|\leq OPT\cdot \left(\ln\frac{k\cdot n}{OPT}+1\right)\leq OPT\cdot \left(\ln\left(\frac{k\cdot n}{OPT_{LP}}\right)+1\right).$$

Hence, the approximation ratio of GreedyDLDT is upper bounded by $\left(\ln\frac{k\cdot n}{OPT_{LP}}+1\right)$. ☐

According to the constraint defined in Equation (2) for the LP problem, we have $OPT_{LP}\geq k$, and $\ln\frac{k\cdot n}{OPT_{LP}}+1\leq \ln n+1$. Therefore, Theorem 3 provides a tighter upper bound than the one in Theorem 2.

### 4.2. Algorithm Design for DLCT

In DLCT, the candidate sensor locations are given as a set of discrete points, and the surfaces of the target objects are composited by a finite set of continuous 2D areas, in which, any point must be *k*-covered. Obviously, a continuous area contains an infinite number of points, which makes it infeasible to provide a direct formulation of this problem as an ILP task. In addition, there lack efficient tools to tackle such complex geometric objects. In this section, we propose a discretization approach to discretize a continuous target area into divisions in order to convert DLCT to DLDT without the loss of precision such that every point in each continuous target area is *k*-covered. The discretization process takes three steps: (i) intersected circle calculation; (ii) division calculation; and (iii) covering set calculation.

#### 4.2.1. Intersected Circle Calculation

The first step for intersected circle calculation is to compute the sensing region within a continuous target area t∈T of a sensor placed at a discrete sensor location l∈L. We first draw a 3D sphere of radius $r_{s}$ centered at the sensor location *l*, and then compute the circle (or arc) intersected between the sphere and the plane where the continuous target area *t* resides. This step is of time complexity O(m·n). All points inside the intersected circle in the target area *t* must be covered by the sensor location *l*. Note that a sphere may intersect with one or more target areas, while a target area may also be intersected by multiple spheres. Therefore, there may be multiple intersected circles or arcs of different radiuses that divide the continuous target area *A* into a number of subareas, referred to as divisions. Note that a division contains at least two intersection points and is enclosed by at least two arcs.

**Definition** **3.** *An* intersection point *is either an internal point (inside area A) intersected by two or more circles, or a border point intersected by the border of area A and one or more circles.*

**Definition** **4.** *A* point of tangency *is either an internal point (inside area A), at which two or more circles are tangent, or a border point, at which the border of area A is tangent to one or more circles. Here, we do not consider a point of tangency as an intersection point.*

**Definition** **5.** *An* arc *is a minimum segment bounded by two intersection points (endpoints) on the rim of a circle. Here, “minimum" means that there does not exist any intersection point between two endpoints.*

**Definition** **6.** *A* division *is a minimum enclosed area bounded by a set of arcs or the segments of the boundary of a continuous target area. Here, “minimum" means that there does not exist any arc inside a division, or a division cannot be divided by any arc into smaller divisions.*

#### 4.2.2. Division Calculation

The second step for division calculation is to determine all divisions formed by the intersected circles calculated at the previous step. This problem appears to be computationally challenging in geometry, but it turns out that tracing all possible divisions can be done in polynomial time. In fact, Huang  et al. pointed out that the total number of intersected divisions could be as many as $O(m^{2})$, where *m* denotes the number of circles, but they did not provide a proof [15]. Here , we provide a rigorous derivation for a tight upper bound on the total number of divisions intersected by *m* circles in an infinite 2D plane. We shall start from the following lemmas.

**Lemma** **1.** Given m circles in an infinite 2D plane, if a newly added circle maximizes the total number of divisions, this new circle must not pass any intersection point created by the existing m circles.

**Proof.**  We prove this lemma by reduction to absurdity. Assume that a newly added circle passes an intersection point *p*, which is created by the intersection of some existing circles, and produces the maximum number of divisions. We use *o* to denote the center of the newly added circle. If we move this circle towards the direction $\vec{po}$ or $\vec{op}$ for a small distance of ε (ε>0 is an arbitrarily small value) without passing any other intersection points, we are able to increase the number of divisions at least by 1, which is in conflict with the assumption. Hence, the newly added circle should not pass any existing intersection point. ☐

**Lemma** **2.** The number of divisions intersected by m circles in an infinite 2D plane is upper bounded by $\left(m^{2}-m+1\right)$.

**Proof.**  We prove this theorem using mathematical induction. Let $P\left(m\right)=\left(m^{2}-m+1\right)$. In the base case where m=2, we have P(2)=3 and there are at most 3 divisions created by 2 circles. Obviously, the theorem holds in this base case.

Suppose that P(m) holds. Now we show that P(m+1) also holds. We first prove that adding a new circle $c^{\prime }$ to the existing *m* circles $\left\{c_{1},c_{2},\ldots ,c_{m}\right\}$ would increase the number of divisions by at most 2m, assuming that $c^{\prime }$ intersects with all existing circle $c_{i}$, i∈[1,m]. Obviously, the number of new intersection points on any existing circle $c_{i}$ is at most 2, and the increment of arcs on $c_{i}$ is also at most 2. Since an arc is shared by at most two divisions (on both sides), the increment of divisions consisting of 2 new arcs on $c_{i}$ is at most 4. Therefore, the total increment of divisions is at most 4m. Since each new division contains at least one arc from $c_{i}$, all new divisions have been counted in the division increment of 4*m* including those covered by $c^{\prime }$ only. However, the above calculation still contains some duplicated divisions, which are addressed as follows.

According to Lemma 1, $c^{\prime }$ only intersects the arcs on $c_{i}$, and $c^{\prime }$ does not go through any existing intersection points on $c_{i}$. The intersection between $c^{\prime }$ and $c_{i}$ falls into one of the following two cases: Case 1 where $c^{\prime }$ intersects only one arc of $c_{i}$, and Case 2 where $c^{\prime }$ intersects two arcs of $c_{i}$.

**Case 1: $c^{\prime }$ intersects only one arc of $c_{i}$**

**Figure 1:**
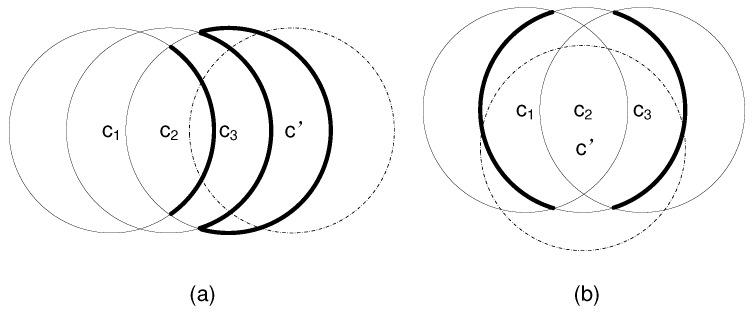
Illustration of two intersection cases between a new circle and the existing circles: (**a**) the new circle $c^{\prime }$ intersects only one arc of an existing circle; (**b**) the new circle $c^{\prime }$ intersects two arcs of an existing circle.

In this case, an arc of $c_{i}$ is divided into three arcs by $c^{\prime }$. An example is shown in Figure 1a, where a boldly marked arc of $c_{1}$, $c_{2}$, or $c_{3}$ is divided into three new arcs by $c^{\prime }$ (marked by dashed lines). We refer to the original arc as $arc_{0}\left(c_{i}\right)$, and the three new arcs as $arc_{1}\left(c_{i}\right)$, $arc_{2}\left(c_{i}\right)$, and $arc_{3}\left(c_{i}\right)$ in the clockwise direction, respectively. We consider the following two intersection conditions: (i) $c^{\prime }$ does or does not intersect an arc of $c_{j_{1}}$ ($j_{1}\in \left[1,m\right],j_{1}\neq i$) inside $c_{i}$; (ii) $c^{\prime }$ does or does not intersect an arc of $c_{j_{2}}$ ($j_{2}\in \left[1,m\right],j_{2}\neq i$) outside $c_{i}$. By combining the above two intersection conditions, we have the following 4 subcases of intersection.

- Subcase (1): $c^{\prime }$ intersects an arc of $c_{j_{1}}$ ($j_{1}\in \left[1,m\right]$, $j_{1}\neq i$) inside $c_{i}$ and also intersects an arc of $c_{j_{2}}$ ($j_{2}\in \left[1,m\right],j_{2}\neq i$) outside $c_{i}$. This is the case for $arc_{0}\left(c_{2}\right)$ in Figure 1a. Before adding $c^{\prime }$, there are 2 divisions sharing $arc_{0}\left(c_{i}\right)$: one division $div_{1}\left(c_{i}\right)$ inside $c_{i}$, and the other division $div_{2}\left(c_{i}\right)$ outside $c_{i}$. After adding $c^{\prime }$, $arc_{0}\left(c_{i}\right)$ is divided into 3 new arcs, and both $div_{1}\left(c_{i}\right)$ and $div_{2}\left(c_{i}\right)$ are divided into 3 new divisions by $c^{\prime }$, resulting in total 6 new divisions, each of which contains one of these 3 new arcs. Therefore, the increment of divisions that share the new arcs of $c_{i}$ is 6−2=4. For each of these 6 new divisions, it consists of at least two new arcs: one new arc from $c_{i}$ and the other new arc from $c_{j_{1}}$ or $c_{j_{2}}$. Obviously, each of these 6 new divisions is duplicately counted by $c_{i}$ and $c_{j_{1}}$ or $c_{j_{2}}$. Therefore, the average increment of divisions sharing the new arcs of $c_{i}$ is 4/2 = 2.
- Subcase (2): $c^{\prime }$ intersects an arc of $c_{j_{1}}$ ($j_{1}\in \left[1,m\right]$, $j_{1}\neq i$) inside $c_{i}$ but does not intersect any arc of $c_{j_{2}}$ ($j_{2}\in \left[1,m\right],j_{2}\neq i$) outside $c_{i}$. This is the case for $arc_{0}\left(c_{3}\right)$ in Figure 1a. Before adding $c^{\prime }$, there is one division $div_{1}\left(c_{i}\right)$ sharing $arc_{0}\left(c_{i}\right)$ inside $c_{i}$, and there may or may not exist a division outside $c_{i}$. After adding $c^{\prime }$, $arc_{0}\left(c_{i}\right)$ is divided into 3 new arcs, and $div_{1}\left(c_{i}\right)$ is divided into 3 new divisions. Therefore, the increment of divisions inside $c_{i}$ sharing the new arcs of $c_{i}$ is 3−1=2. For each of these 3 new divisions inside $c_{i}$, it contains at least two new arcs: one new arc from $c_{i}$ and the other new arc from $c_{j_{1}}$. Hence, the average increment of divisions inside $c_{i}$ sharing the new arcs of $c_{i}$ is 2/2 = 1. Whether or not there exists a division $div_{2}\left(c_{i}\right)$ outside $c_{i}$ before adding $c^{\prime }$, the increment of divisions outside $c_{i}$ is always 1. Therefore, the average increment of divisions both inside and outside $c_{i}$ sharing the new arcs of $c_{i}$ is 1 + 1 = 2.
- Subcase (3): $c^{\prime }$ does not intersect any arc of $c_{j_{1}}$ ($j_{1}\in \left[1,m\right]$, $j_{1}\neq i$) inside $c_{i}$ but intersects an arc of $c_{j_{2}}$ ($j_{2}\in \left[1,m\right],j_{2}\neq i$) outside $c_{i}$. This is the case for $arc_{0}\left(c_{1}\right)$ in Figure 1a. Similar to the analysis for Subcase (2), the average increment of divisions both inside and outside $c_{i}$ sharing the new arcs of $c_{i}$ is 2.
- Subcase (4): $c^{\prime }$ does not intersect any arc of $c_{j_{1}}$ ($j_{1}\in \left[1,m\right]$, $j_{1}\neq i$) inside $c_{i}$ nor intersects any arc of $c_{j_{2}}$ ($j_{2}\in \left[1,m\right],j_{2}\neq i$) outside $c_{i}$. An example of this case would be to add $c^{\prime }$ that intersects only one existing circle. Since the increment of divisions either inside or outside $c_{i}$ is 1, the average increment of divisions both inside and outside $c_{i}$ is 2.

**Case 2: $c^{\prime }$ intersects two arcs of $c_{i}$**

In this case, two arcs of $c_{i}$ are divided into two new arcs by $c^{\prime }$, respectively. An example is shown in Figure 1b, where each of the two boldly marked arcs of $c_{2}$ is divided into two new arcs by $c^{\prime }$ (marked by dashed lines). We consider one of the original arcs and refer to it as $arc_{0}\left(c_{i}\right)$. Similar to Case 1, we consider 4 subcases with different combinations of the following two intersection conditions: $c^{\prime }$ does or does not intersect an arc of $c_{j_{1}}$ ($j_{1}\in \left[1,m\right],j_{1}\neq i$) inside $c_{i}$ and $c^{\prime }$ does or does not intersect an arc of $c_{j_{2}}$ ($j_{2}\in \left[1,m\right],j_{2}\neq i$) outside $c_{i}$. In each subcase, the average increment of divisions both inside and outside $c_{i}$ sharing the new arcs (only from $arc_{0}\left(c_{i}\right)$) of $c_{i}$ is 1. Since there are two original arcs, the total average increment of divisions both inside and outside $c_{i}$ sharing the new arcs of $c_{i}$ is 2.

In sum, the average increment of divisions for any existing circle $c_{i}$ is 2. Since there are total *m* existing circles, the total increment of divisions is 2m. It follows that $P\left(m+1\right)=P\left(m\right)+2m={\left(m+1\right)}^{2}-\left(m+1\right)+1$. Therefore, P(m+1) holds. Since both the base and inductive steps are validated, we conclude that Lemma 2 holds for all natural number *m* according to the theory of mathematical induction. ☐

According to Lemma 1, *m* circles should be placed in such a way that every intersection point is created by only two circles in order to maximize the number of divisions. We show one tight example of Lemma 2 in Figure 1, where the centers of all these circles of an identical radius are aligned along a line, and every newly added circle intersects all existing circles.

We can represent a division using a sequence of component intersection points and arcs. To facilitate division calculation, we provide below several definitions.

**Definition** **7.** $end_{1}\left(arc\right)$ and $end_{2}\left(arc\right)$ are the endpoints of an arc in the clockwise direction, and mid(arc) is the middle point of an arc.

**Definition** **8.** The tangent of an arc at one endpoint p is defined as a tangent radial that is tangent to the arc at p, and the angle between the tangent radial and the chord $\vec{p,mid(arc)}$ is less than 90 degrees.

**Definition** **9.** Suppose that arc $arc_{1}$ and arc $arc_{2}$ share one endpoint p. The angle from arc $arc_{2}$ to arc $arc_{1}$ is defined as the angle from the tangent of $arc_{2}$ at its endpoint p to the tangent of $arc_{1}$ at its endpoint p in the clockwise direction.

**Figure 2:**
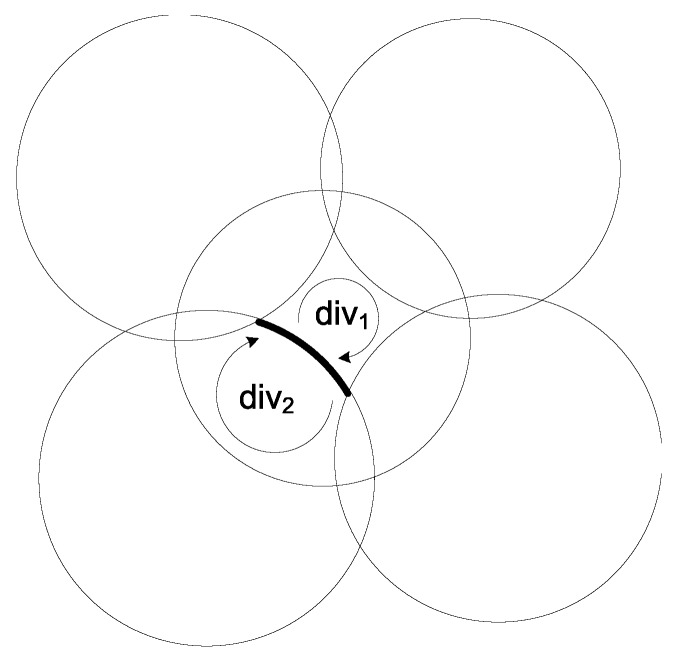
An example of Algorithm 2.

We design an algorithm in Algorithm 2 to determine all divisions in a continuous area *A* intersected by *m* circles and the boundary $\bar{A}$ of *A*. In lines 5–6, it adds a new circle, which intersects with neither any other circles nor the boundary $\bar{A}$, or is only tangent to one circle or $\bar{A}$. In lines 10–18, it traces a division outside the circle or *A*, to which $arc_{src}$ belongs. In lines 19–27, it traces a division that is inside the circle or *A*, to which $arc_{src}$ belongs. It returns all possible divisions inside the continuous area *A*. The computational complexity of Algorithm 2 is $O(m^{3})$ in the worst case. An example of Algorithm 2 is illustrated in Figure 2, where $arc_{src}$ is represented by a boldly marked arc, and the divisions on both sides of $arc_{src}$ are denoted by $div_{1}$ and $div_{2}$. The curved arrows show the tracing directions we follow to identify these two divisions.
**Algorithm 2** Division Calculation **Input:** A continuous area *A*, *m* circles $\left\{c_{1},c_{2},\ldots ,c_{m}\right\}$ on *A*. **Output:** All divisions within *A*.1:$Cirs=\{c_{1},c_{2},\ldots ,c_{m}\},Arcs=\emptyset ,Divs=\emptyset ,DivCmps=\emptyset$;2:Calculate all the intersection points on $c_{i}\in Cirs$ and $\bar{A}$, and record the list of intersection points on $c_{i}$;3:Add all the arcs in $\bar{A}$ to Arcs if $\bar{A}$ has intersection points;4:**for all** $c_{i}\in Cirs$ **do**5: **if** $c_{i}$ has no intersection point and is inside *A* **then**6:  Add $c_{i}$ to DivCmps and then to Divs;7: **else**8:  Add all arcs on $c_{i}$ and inside *A* to Arcs;9:**for all** $arc_{src}\in Arcs-\bar{A}$ **do**10: $arc_{cur}=arc_{src}$;11: **repeat**12:  Select $arc_{next}\in Arcs$ such that $arc_{next}$ share the endpoint $end_{1}\left(arc_{cur}\right)$ and the angle from $arc_{next}$ to $arc_{cur}$ is minimized;13:  $arc_{cur}$ = $arc_{next}$;14: **until** $(arc_{cur}$ == $arc_{src})$15: **if** the chain $d_{1}$ of arcs form the profile of multiple circles **then**16:  Add $d_{1}$ to DivCmps if $d_{1}\notin DivCmps$;17: **else**18:  Add $d_{1}$ to Divs if $d_{1}\notin Divs$;19: $arc_{cur}=arc_{src}$;20: **repeat**21:  Select $arc_{next}\in Arcs$ such that $arc_{next}$ share the endpoint $end_{2}\left(arc_{cur}\right)$ and the angle from $arc_{next}$ to $arc_{cur}$ is minimized;22:  $arc_{cur}$ = $arc_{next}$;23: **until** $(arc_{cur}$ == $arc_{src})$24: **if** the chain $d_{2}$ of arcs form the profile of multiple circles **then**25:  Add $d_{2}$ to DivCmps if $d_{2}\notin DivCmps$;26: **else**27:  Add $d_{2}$ to Divs if $d_{2}\notin Divs$;28:**if** there is no intersection point in $\bar{A}$ **then**29: **if** Divs≠∅ or *A* is inside a circle **then**30:  Add $\bar{A}$ to Divs;31:**for all** $d_{i}\in DivCmps$ **do**32: $d_{min}=\emptyset$;33: **for all** $d_{j}\in Divs$ **do**34:  **if** $d_{j}$ encloses $d_{i}$ and ($d_{min}=\emptyset$ or *d* encloses $d_{j}$) **then**35:   $d_{min}=d_{j}$;36: Incorporate $d_{i}$ into $d_{min}$ and update $d_{min}$ in Divs;37:**return** Divs.

We use Algorithm 2 to determine all divisions in each continuous target area t∈T divided by the intersected circles resulted from the first step and the boundaries of *t*. With *n* continuous targets, division calculation is of time complexity $O(m^{3}\cdot n)$.

#### 4.2.3. Covering Set Calculation

The third step for covering set calculation is to determine the set of divisions a discrete sensor location l∈L can cover. A discrete sensor location may cover the divisions from multiple continuous targets, and may only cover a subset or the entire set of divisions from one target. Note that a continuous target is *k*-covered if and only if every division within the target area is *k*-covered. The following lemma is used to check if a sensor location can cover a division, and compute the covering set of each sensor location l∈L. This step is of time complexity $O(m^{3}\cdot n)$.

**Lemma** **3.** A discrete sensor location covers an arc if and only if the distance from each endpoint of the arc to the sensor location is less than or equal to $r_{s}$ and the distance from the middle point of the arc to the sensor location is also less than or equal to $r_{s}$. A discrete sensor location covers a division if and only if it covers all the component arcs of the division.

If a division contains a non-arc boundary segment of a target area, then the segment is covered by a sensor if and only if the distance from each endpoint of the segment to the sensor location is less than or equal to $r_{s}$ and the segment does not intersect with the covering circle of the sensor. If a division is constituted by a single circle, we evenly divide the circle into two arcs and apply the above lemma to check if a sensor covers a circular division.

The complexity of the entire discretization process is dominated by the complexity of the last two steps, i.e., $O(m^{3}\cdot n)$. If we treat every target division as a discrete virtual target point, we are able to convert DLCT to DLDT without the loss of any precision. Therefore, we adapt GreedyDLDT for DLDT to DLCT, referred to as GreedyDLCT, after discretizing continuous target areas, and prove that the performance of this adapted approach to DLCT is upper bounded by $\left(\ln nm^{2}+1\right)$.

**Theorem** **4.** The approximation ratio of GreedyDLCT with target object discretization for DLCT is $\left(\ln nm^{2}+1\right)$.

**Proof.**  We compute the maximum total number of divisions in all continuous target areas. Since every continuous target object is a convex area, Lemma 2 shows that the maximum number of divisions in one continuous target area is $\left(m^{2}-m+1\right)$. With *n* continuous target areas, the maximum total number of divisions in all these areas is $n\cdot (m^{2}-m+1)$. According to Equation (8), we have

(10) $$\begin{array}{cc} |L_{0}|\leq & OPT\cdot (\ln\frac{k\cdot n\cdot (m^{2}-m+1)}{OPT}+1)\\ \leq & OPT\cdot (\ln\frac{k\cdot n\cdot m^{2}}{OPT}+1)\\ \leq & OPT\cdot (\ln nm^{2}+1). \end{array}$$

Hence, the approximation ratio of GreedyDLCT is $\left(\ln nm^{2}+1\right)$. ☐

### 4.3. Algorithm Design for CLDT

In CLDT, the feasible sensor locations are given as a set of 2D continuous areas, and the target objects are given as a set of discrete points. We may place sensors at any point within these continuous areas for sensor deployment. The key idea for solving CLDT is to use a similar three-step discretization process to discretize a continuous sensor location into divisions in order to convert CLDT to DLDT without loss of precision, referred to as GreedyCLDT.

Similar to DLCT, during discretization in CLDT, the first step for intersected circle calculation is to compute the valid sensor region within each continuous feasible sensor location l∈L that covers a discrete target point t∈T. We first draw a 3D sphere of radius $r_{s}$ centered at a target point *t*, and then compute the circle or arc intersected by the sphere and the continuous sensor location *l*. If a sensor is placed within the intersected circle inside the sensor location *l*, it must cover *t*. In addition, a sphere may intersect one or more sensor locations, and a feasible sensor location may also be intersected by multiple spheres. Multiple intersected circles or arcs of different radiuses may exist at every feasible sensor location.

**Figure 3:**
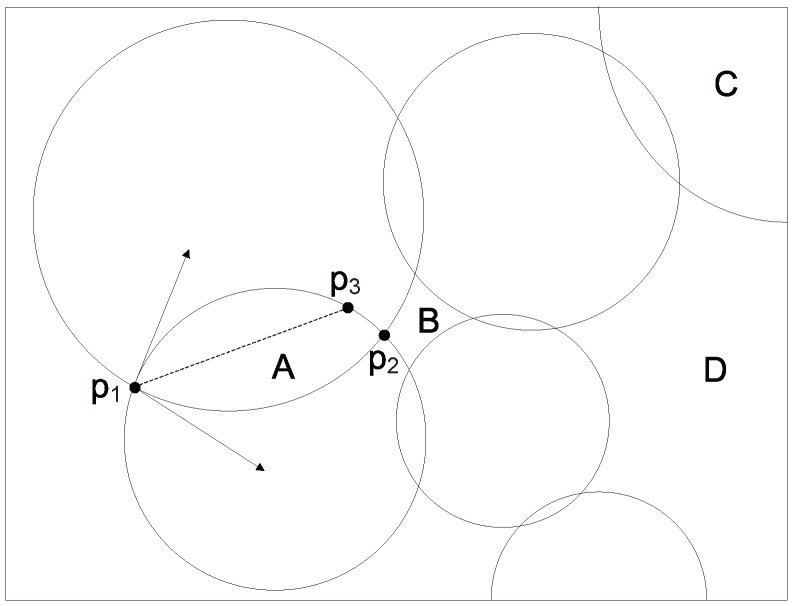
Intersected circles and divisions.

The second step for division calculation is to use Algorithm 2 to determine all possible divisions at every continuous sensor location l∈L divided by the intersected circles resulted from the first step and the boundaries of *l*. Note that a valid division must be inside an intersected circle such that a sensor deployed within this division can cover at least one discrete target point. However, not all the divisions returned by Algorithm 2 are valid. We show one division example in Figure 3, where the surrounding rectangle is a continuous sensor location, and it is divided into a number of divisions by the intersected circles or arcs. In Figure 3, divisions A and C are valid, while divisions B and D are invalid. We first compute all divisions at every sensor location l∈L, and then remove any invalid divisions. Every valid division can be treated as a discrete candidate sensor location because we can place a sensor at any point within the division while achieving the same coverage performance. Obviously, we can deploy at most *k* sensors within a valid division.

The second step for division calculation is to use Algorithm 2 to determine all possible divisions at every continuous sensor location l∈L divided by the intersected circles resulted from the first step and the boundaries of *l*. Note that a valid division must be inside an intersected circle such that a sensor deployed within this division can cover at least one discrete target point. However, not all the divisions returned by Algorithm 2 are valid. We show one division example in Figure 3, where the surrounding rectangle is a continuous sensor location, and it is divided into a number of divisions by the intersected circles or arcs. In Figure 3, divisions A and C are valid, while divisions B and D are invalid. We first compute all divisions at every sensor location l∈L, and then remove any invalid divisions. Every valid division can be treated as a discrete candidate sensor location because we can place a sensor at any point within the division while achieving the same coverage performance. Obviously, we can deploy at most *k* sensors within a valid division.

We choose *k* discrete points within a division as follows. Let $arc_{1}$ and $arc_{2}$ be two arcs of a division that share an intersection point *p*. We compute the tangents of arcs $arc_{1}$ and $arc_{2}$ at point *p*, respectively, and the bisector of the angle formed by these two tangents. Suppose that the angle bisector intersects one of the arcs of the division at point *q*. We can evenly lay out *k* points along line segment $\bar{pq}$, which are used to represent the division. All the sensors placed at these *k* points within the same division have an identical coverage performance. We provide an example of computing *k* points within a division in Figure 3, where we use *k* points on line segment $\bar{p_{1}p_{3}}$ to represent division A. We calculate *k* discrete points within every valid division at each sensor location l∈L.

The third step for covering set calculation is to compute the set of discrete target points that every discrete point resulted from the previous step can cover. The above three-step discretization process converts CLDT to DLDT, and the time complexity of this transformation is $O(n^{3}\cdot m\cdot k)$. We then apply GreedyDLDT to compute a solution to CLDT. As the number of target points remains the same, the approximation ratio of GreedyCLDT is (lnn+1).

### 4.4. Algorithm Design for CLCT

In CLCT, both sensor locations and target objects are given as continuous 2D areas. This problem is more challenging to tackle because we can not directly convert it to DLDT using the same discretization method for DLCT and CLDT. We propose to first divide CLCT into two subproblems, and then convert the two subproblems to CLDT and DLCT, respectively. Based on this approach, we design an approximate algorithm for CLCT.

#### 4.4.1. Problem Decomposition

Let *S* denote the enclosed 3D space that is collectively covered by all sensor locations with sensing radius $r_{s}$ and $\bar{S}$ denote the surface of *S*.

There are four cases in the CLCT problem.

- Case 1: At least a target point exists outside *S*. In this case, there is no feasible solution to the CLCT problem, and hence is ignored.
- Case 2: No target point is outside *S*, but $\bar{S}$ and target areas have intersected points whose neighbourhoods within the target areas can not be covered by finite sensor location points. In this case, the minimal number of selected sensor location points to cover all the targets is infinitely large. Therefore, we do not need to consider this case.
- Case 3: No target point is outside *S*, and the neighbourhood of any point intersected by $\bar{S}$ and a target area can be covered by finite sensor location points. In this case, for each sensor location *l* covering a target point in $\bar{S}$, we draw a sphere of radius $r_{s}$ centered at *l*. A target area may be intersected by multiple spheres. In every target area, there may be multiple intersected arcs that divide the target area into several divisions. Then, temporarily remove all the divisions adjacent to a target point in $\bar{S}$. The minimum distance between $\bar{S}$ and all the points in the rest target areas is denoted by *d* (d>0).
- Case 4: All the target areas are in the inner side of *S*. In this case, there is no target point in $\bar{S}$. The minimal distance between $\bar{S}$ and the points in the target areas is denoted by *d* (d>0).

Let $S_{1}$ represent the enclosed 3D space that is collectively covered by all sensor locations with sensing radius $\left(r_{s}-d\right)$. Let $S_{2}=S-S_{1}$. Then, we divide the CLCT problem into two subproblems as follows:

- Subproblem 1: Given *m* convex sensor location areas and convex target areas in $S_{1}$, deploy a minimum number of sensors with sensing radius $r_{s}$ to *k*-cover all the target areas in $S_{1}$, referred to as $SubOPT_{1}$.
- Subproblem 2: Given *m* convex sensor location areas and continuous target areas in $S_{2}$, deploy a minimum number of sensors with sensing radius $r_{s}$ to *k*-cover all the target areas in $S_{2}$, referred to as $SubOPT_{2}$.

Note that in Case 4, the CLCT problem is the same as Subproblem 1.

#### 4.4.2. Coverage over Internal Targets

We first tessellate 3D region *R* by cubes with edge of $a=d/\sqrt{3}$. Each target area may cut some cubes. In each cut cube, we arbitrarily select a point in a target area in $S_{1}$, and obtain $n_{1}$ discrete target points. Then, we construct two CLDT problems.

- Problem 1: Given *m* convex sensor location areas and $n_{1}$ target points, deploy a minimum number of sensors with sensing radius $r_{s}$ to *k*-cover $n_{1}$ target points, referred to as $CLDTOPT_{1}$.
- Problem 2: Given *m* convex sensor location areas and $n_{1}$ target points, deploy a minimum number of sensors with sensing radius $\left(r_{s}-d\right)$ to *k*-cover $n_{1}$ target points, referred to as $CLDTOPT_{2}$.

Problem 2 is a CLDT problem with an approximation algorithm, so that we can use the approximate solution $CLDTAS_{2}$ to Problem 2 as the approximate solution $SubAS_{1}$ to Subproblem 1.

**Definition** **10.** **Critical sensor location points**: All the sensor location points that can cover a target point in $\bar{S}$ are referred to as a set $L_{c}$ of critical sensor location points.

In Problem 2, if selected location divisions contain a point in $L_{c}$, the location point will be selected to represent the location division.

**Lemma** **4.** The approximation ratio of the algorithm for Subproblem 1 is $f\left(\frac{r_{s}}{d}\right)\left(\ln n+1\right)$, where $f\left(p\right)=36\sqrt{3}\pi {\left(p-\frac{1}{2}\right)}^{2}+27\sqrt{3}\pi +1$.

**Proof.**  Obviously, a set of sensors *k*-covering all the target areas in $S_{1}$ can *k*-cover $n_{1}$ target points. In addition, if a set of sensors with sensing radius $\left(r_{s}-d\right)$ that can *k*-cover $n_{1}$ target points have sensing radius $r_{s}$, they can also *k*-cover all the target areas in $S_{1}$. Hence, $|CLDTOPT_{1}|\leq |SubOPT_{1}|\leq |CLDTOPT_{2}|$.

Since the approximation ratio of GreedyCLDT is (lnn+1),

(11) $$\frac{|SubAS_{1}|}{|SubOPT_{1}|}\leq \frac{|CLDTAS_{2}|}{|CLDTOPT_{1}|}\leq \frac{|CLDTOPT_{2}|\cdot \left(\ln n+1\right)}{|CLDTOPT_{1}|}.$$

Since we can deploy $|CLDTOPT_{1}|$ sensors with sensing radius $r_{s}$ to *k*-cover all the discrete target points, we firstly deploy $|CLDTOPT_{1}|$ sensors with sensing radius $\left(r_{s}-d\right)$ in the same locations. For each sensor location, only target points between the two concentric spheres with radii $r_{s}$ and $\left(r_{s}-d\right)$ lose a coverage due to the decrease of the sensing radius. Fortunately, the number of target points in the region has an upper bound, denoted by *l*, in that there is at most one target point in a cube with edge *a*. In other words, each sensor causes no more than *l* target points to lose a coverage. Totally, $l\cdot |CLDTOPT_{1}|$ coverage disappears. Then, additional $l\cdot CLDTOPT_{1}$ sensors are sufficient to provide *k*-coverage over all the target points. Therefore, we can deploy no more than $\left(l+1\right)\cdot |CLDTOPT_{1}|$ sensors with sensing radius $\left(r_{s}-d\right)$ to *k*-cover all the target points, i.e., $|CLDTOPT_{2}|\leq \left(l+1\right)\cdot |CLDTOPT_{1}|$.

(12) $$\frac{|CLDTAS_{2}|}{|SubOPT_{1}|}\leq \frac{|CLDTOPT_{2}|\cdot \left(\ln n+1\right)}{|CLDTOPT_{1}|}\leq \left(l+1\right)\cdot \left(\ln n+1\right).$$

We provide the upper bound *l*. On one hand, all the cubes that can be intersected by a full sphere with radius $r_{s}$ can be completely contained in a sphere with radius $\left(r_{s}+d\right)$, so there are at most 43π(rs+d)343π(rs+d)3dd333dd333=43πrsd+13 cubes. On the other hand, the 3D region composed of all the cubes that can necessarily be contained but not intersected by a sphere with radius $\left(r_{s}-d\right)$ regardless of their locations relative to the sphere can contain a sphere with radius $\left(r_{s}-2d\right)$, so the number of cubes is at least 43π(rs−2d)343π(rs−2d)3dd333dd333=43πrsd−23. Hence,

(13) $$l\leq 4\sqrt{3}\pi {\left(\frac{r_{s}}{d}+1\right)}^{3}-4\sqrt{3}\pi {\left(\frac{r_{s}}{d}-2\right)}^{3}.$$

According to Equations (12) and (13), we have

(14) $$\frac{|CLDTAS_{2}|}{|SubOPT_{1}|}\leq \left[36\sqrt{3}\pi {\left(\frac{r_{s}}{d}-\frac{1}{2}\right)}^{2}+27\sqrt{3}\pi +1\right]\cdot \left(\ln n+1\right).$$

☐

#### 4.4.3. Coverage over Targets on the Border

We construct a DLCT problem as follows: Given a set $L_{c}$ of sensor location points and target areas in $S_{2}$, deploy a minimum number of sensors with sensing radius $r_{s}$ at location points in $L_{c}$ to *k*-cover all the target areas in $S_{2}$, referred to as DLCTOPT.

**Lemma** **5.** $|DLCTOPT|=|SubOPT_{2}|$.

**Proof.**  Consider the DLCT problem. For each sensor location *l* in $L_{c}$, draw a sphere with radius $r_{s}$ centered at *l*. All the spheres and all the target areas in $S_{2}$ may intersect at multiple arcs. The arcs divide the target areas in $S_{2}$ into some divisions, which are all adjacent to at least a target point in $\bar{S}$. Assume that there exist a division that can be covered by a set $L_{1}$ of finite sensor locations in $L-L_{c}$. The division is adjacent to the target point *t* in $\bar{S}$. Let $d^{\prime }$ be the minimum distance between *t* and all the sensor location points in $L_{1}$. Since $d^{\prime }>d$, the target points less than $\left(d^{\prime }-d\right)$ far from *t* in the division cannot be covered by any points in $L_{1}$, which contradicts with the assumption that the division can be covered by $L_{1}$. Hence, no division can be covered by finite sensor locations in $L-L_{c}$.

Based on the definition of $S_{2}$, all the location points in $L_{c}$ can collectively cover all the target areas in $S_{2}$, so the DLCT problem has at least a feasible solution. Since  $L_{c}\subseteq L$, $|DLCTOPT|\geq |SubOPT_{2}|$. Assume that $|DLCTOPT|>|SubOPT_{2}|$. Then, there exists at least one sensor in (DLCTOPT−SubOPT). If the sensors in (DLCTOPT−SubOPT) are all moved out of DLCTOPT, there must be a target division, in which no internal point is *k*-covered by $SubOPT_{2}\cap L_{c}$, because $\left(SubOPT_{2}\cap L_{c}\right)\subseteq L_{c}$ and $|SubOPT_{2}\cap L_{c}\left|<|DLCTOPT\right|$. Since $SubOPT_{2}$ is a feasible solution, the entire target division can be covered by no more than $|SubOPT_{2}|$ sensors located at $L-L_{c}$, which contradicts with the fact that the finite sensor location points in $L-L_{c}$ cannot cover any target division in $S_{2}$. It follows that $|DLCTOPT|\leq |SubOPT_{2}|$. Hence, $|DLCTOPT|=|SubOPT_{2}|$. ☐

By converting Subproblem 2 to DLCT that is solved by an approximation algorithm, we can obtain an approximation solution $SubAS_{2}$ to Subproblem 2. In Subproblem 2, if some divisions in the target areas have been completely covered by the set of sensors selected in Subproblem 1, the number of required covers over the divisions can be reduced correspondingly in order to decrease the number of deployed sensors.

There are only two existing forms for the points intersected by $\bar{S}$ and target areas:

- Case 1: an isolated point in $\bar{S}$.
- Case 2: a set of concyclic points, all of which only one or two sensor location points can cover.

If all the target areas can be *k*-covered by finite sensors, the total number of isolated points and sets of concyclic points intersected by $\bar{S}$ and target areas must be a finite number, denoted by $n_{2}$.

**Lemma** **6.** The approximation ratio of the greedy algorithm for Subproblem 2 is $\left(\ln mn_{2}^{2}+1\right)$, where m is the number of sensor location areas.

**Proof.**  Assume that in a sensor location area, there exist two location points $l_{1}$ and $l_{2}$ covering the same target point *t* in $\bar{S}$. Then, their distances to *t* are $\left|\bar{l_{1}t}\right|=\left|\bar{l_{2}t}\right|=r_{s}$. Because the sensor location area is convex, any point *l* on the line segment $\bar{l_{1}l_{2}}$ is in the location area. Since $l\in \bar{l_{1}l_{2}}$ and $\left|\bar{l_{1}t}\right|=\left|\bar{l_{2}t}\right|=r_{s}$, the distance $\left|\bar{lt}\right|$ is less than the sensing distance $r_{s}$, which contradicts with the assumption of $t\in \bar{S}$. Therefore, for each target point in $\bar{S}$, there is at most one valid location point in a sensor location area. Since the total number of isolated target points and sets of concyclic target points in $\bar{S}$ is no more than $n_{2}$, $\left|L_{c}\right|\leq m\cdot n_{2}$. According to Theorem 4, the approximation ratio of the greedy algorithm for Subproblem 2 is $\left(\ln mn_{2}^{2}+1\right)$. ☐

#### 4.4.4. Approximation Ratio

**Theorem** **5.** The approximation ratio of the algorithm for CLCT is $\left[36\sqrt{3}\pi {\left(\frac{r_{s}}{d}-\frac{1}{2}\right)}^{2}+27\sqrt{3}\pi +1\right]$$\left(\ln n_{1}+1\right)+\ln mn_{2}^{2}+1$.

**Proof.**  Let AS be the approximate solution to the original CLCT problem with optimal solution OPT. We have

(15) $$\frac{\left|AS\right|}{\left|OPT\right|}\leq \frac{|SubAS_{1}|}{\left|OPT\right|}+\frac{|SubAS_{2}|}{\left|OPT\right|}\leq \frac{|SubAS_{1}|}{|SubOPT_{1}|}+\frac{|SubAS_{2}|}{|SubOPT_{2}|}.$$

Based on Equation (15) and Lemma 4 and 6, the approximation ratio of the algorithm for CLCT can be computed as follows:

(16) $$\begin{array}{c} \frac{\left|AS\right|}{\left|OPT\right|}\leq \left[36\sqrt{3}\pi {\left(\frac{r_{s}}{d}-\frac{1}{2}\right)}^{2}+27\sqrt{3}\pi +1\right]\left(\ln n_{1}+1\right)\\ +\ln kmn_{2}^{2}+1. \end{array}$$

 ☐

## 5. Algorithm Design with Connectivity Requirement

We consider another set of four connected deployment problems by imposing a requirement on the sensor network connectivity: C-DLDT, C-DLCT, C-CLDT, and C-CLCT. Obviously, all these problems are still NP-complete since their corresponding version without the connectivity requirement is a special case where every sensor has a sufficiently large communication radius.

We design an approximate algorithm for each of these connected sensor deployment problems. We use C-DLDT as an example to explain the algorithm design, which is based on the result of DLDT returned by Algorithm 1. Suppose that the set of sensor locations from Algorithm 1 for DLDT is $L_{0}$, $L_{0}\subseteq L$. C-DLDT is to select a minimum number of candidate sensor locations from $L-L_{0}$ and add them to $L_{0}$ such that all the sensors in $L_{0}$ are connected, which is similar to the Steiner Tree Problem with Minimum number of Steiner Points and Bounded Edge-Length (STP-MSPBEL) in [24]. They differ in that the candidate Steiner points are limited to a set of given locations in C-DLDT. We define a subproblem, Steiner Tree Problem with Minimum number of Steiner Points at Constrained Locations (STP-MSPCL): given a set *L* of discrete points, a subset $L_{0}$ of *L*, and a communication radius $r_{c}$, the problem is to add a minimum number of points from $L-L_{0}$ to $L_{0}$ such that all the points in $L_{0}$ are connected. Note that two sensors are connected by a wireless link if and only if the Euclidean distance between them is no more than communication radius $r_{c}$. Here, we use $L_{0}$ to denote the set of terminal points and use $L-L_{0}$ to denote the set of Steiner points.

We propose an approximate algorithm for STP-MSPCL based on the minimum spanning tree algorithm in Algorithm 3, which solves the node-weighted Steiner tree problem by reducing it to the edge-weighted Steiner tree problem. In lines 1–9, it assigns a weight to every link. If both of the end points of a link belong to $L_{0}$, then we the weight of this link to be 0; otherwise, we set the weight of this link to be 1. Hence, every connected subset of $L_{0}$ can be treated as a super point, because the length of the path between any two points in the connected subset of $L_{0}$ is 0. In lines 10–13, it computes the shortest path between any two points in $L_{0}$, which is used to build the spanning tree in line 14. Those points that are in the spanning tree but do not belong to $L_{0}$ are Steiner points that have already been added to $L_{0}$ in order to make $L_{0}$ form a connected network. Algorithm 3 is of time complexity $O(m^{3})$ in the worst case.

**Algorithm 3** Approximation Algorithm for STP-MSPCL Input: *L*, $L_{0}$, $r_{c}$ Output: A set *C* of Steiner points, $C\subseteq L-L_{0}$1:**for all** $l_{i},l_{j}\in L$, i≠j **do**2: **if** distance($l_{i},l_{j}$) $\leq r_{c}$ **then**3:  **if** $l_{i}\in L_{0}$ and $l_{j}\in L_{0}$ **then**4:   $w_{\left(l_{i},l_{j}\right)}=0;$5:  **else**6:   $w_{\left(l_{i},l_{j}\right)}=1;$7:**for all** $l_{i},l_{j}\in L_{0}$, i≠j **do**8: Compute the shortest path *P* between $l_{i}$ and $l_{j}$;9: $w_{\left(l_{i},l_{j}\right)}=length\left(P\right)$;10:Compute a minimum spanning tree $MST(L_{0})$ on $L_{0}$;11:Let *C* be the set of locations $l\in L-L_{0}$ on the shortest path between any two locations in $MST(L_{0})$;12:**return** C.

For STP-MSPCL in 2D space where *L* are distributed in a 2D area, we prove that the number of Steiner points in $C\subseteq L-L_{0}$ returned by the approximate algorithm is at most 5 times the number of Steiner points in an optimal solution. In [24], Lin et al. showed that there exists a shortest-length optimal Steiner tree for SMT-MSPBEL such that every Steiner point has a degree of at most 5.

**Theorem** **6.** The approximation ratio of Algorithm 3 for STP-MSPCL in 2D space is 5.

**Proof.**  We first show that the degree of a Steiner point is at most 5. Assume that a Steiner point l∈L has a degree of 6, connecting to 6 terminal points, and the distance between *l* and each of these 6 terminal points is less than or equal to $r_{c}$. There must exist at least two terminal points, the distance between which is less than or equal to $r_{c}$. If the terminal points at most $r_{c}$ away from each other are considered as one single virtual terminal point (since they are connected), a Steiner point can make at most five separated terminal points connected. In our approximate algorithm, every selected Steiner point is on the shortest path between two terminal points. Hence, the number of Steiner points returned by Algorithm 3 is at most five times the number of Steiner points in an optimal solution. ☐

**Figure 4:**
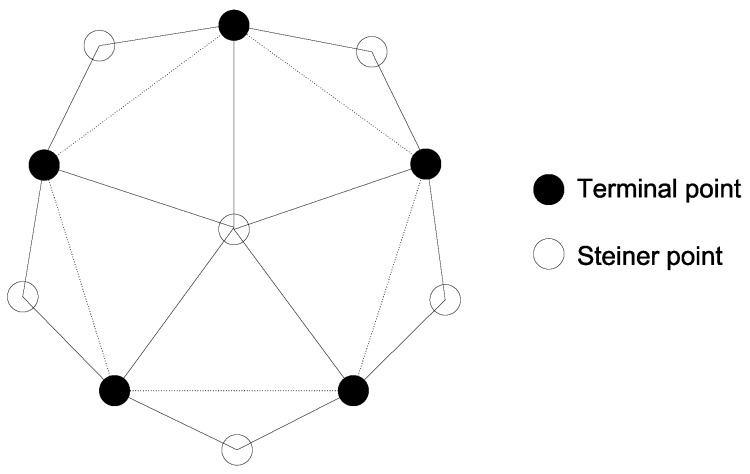
A tight example of Theorem 6.

We provide a tight example of Theorem 6 in Figure 4, where five terminal points are located at the corners of a pentagon (dashed lines) with an edge length greater than $r_{c}$. One Steiner point is located at the center and five other Steiner points are located outside the pentagon. The distance between any two terminal points is greater than $r_{c}$, and the distance from any terminal point to the central Steiner point is less than $r_{c}$. An optimal solution should add the central Steiner point to the set $L_{0}$ of terminal points, but Algorithm 3 may add the other five Steiner points to $L_{0}$.

Before further analyzing STP-MSPCL in 3D space, we would like to describe a long-debated historical “thirteen spheres” problem, which asks if 13 non-overlapping spheres of an identical size can touch the surface of another (central) sphere in 3D space. In a symmetrical configuration, we can place 12 spheres at those positions corresponding to the vertices of a regular icosahedron concentric with the central sphere. As these 12 spheres do not touch each other, there was a conjecture that a 13-th sphere may be added, which was the subject of the famous discussion between Isaac Newton and David Gregory in 1694, and it was finally proved in 1953 that at most 12 spheres can touch the central sphere [25]. For STP-MSPCL where *L* are distributed in 3D space, we shall prove that the number of Steiner points in $C\subseteq L-L_{0}$ returned by the approximate algorithm is at most 12 times the number of Steiner points in an optimal solution.

**Theorem** **7.** The approximation ratio of Algorithm 3 for STP-MSPCL in 3D space is 12.

**Proof.**  We set the radius of each sphere to be $r_{c}/2$ in the “thirteen spheres” problem. We can place at most 12 spheres around the central sphere without touching each other. The distance between the centers of any two outer spheres is greater than $r_{c}$, and the distance from the center of any outer sphere to the center of the central sphere is equal to $r_{c}$. If we deploy a terminal point at the center of each outer sphere and deploy a Steiner point at the center of the central sphere, such a Steiner point can make at most 12 separated terminal points connected. Similar to the proof of Theorem 6, the number of Steiner points returned by the approximate algorithm is at most 12 times the number of Steiner points obtained by an optimal solution. ☐

The algorithm for C-DLDT is a two-stage algorithm: (i) use the approximation algorithm with an approximation ratio of (lnn+1) for DLDT to achieve coverage, and (ii) use the approximation algorithm with an approximation ratio of 12 for STP-MSPCL to achieve connectivity. For $r_{c}\geq 2r_{s}$, this two-stage algorithm for C-DLDT yields a combined approximation ratio of (lnn+1+12)=(lnn+13), which can be proved in a similar way for bounding the approximation ratio of a two-stage algorithm in [26]. We solve the other three connected deployment problems, i.e., C-CLDT, C-DLCT, and C-CLCT, in the same way we solve C-DLDT using a similar two-stage process. Similarly, for $r_{c}\geq 2r_{s}$ in constrained 3D space, the combined approximation ratio of the algorithm for C-DLCT is (ln(k·n·m)+13) and the combined approximation ratio of the algorithm for C-CLDT is (lnn+13).

## 6. Performance Evaluation

We conduct simulation-based performance evaluation on the proposed algorithms for constrained sensor deployment in 3D space using a set of randomly generated problem instances.

We would like to make an emphasis that the proposed methods for sensor deployment are approximate algorithms in nature, which are essentially different from heuristic approaches without any performance bound. In particular, we focus on the performance evaluation of the algorithm designed for DLDT (Algorithm 1) because it serves as the base of the solutions to the other problems, and the performance of this algorithm is a direct reflection of the performance of the other proposed algorithms.

### 6.1. Simulation Setting

In the simulation, we consider a 3D cube of dimensions 100 m × 100 m × 100 m as the region of interest, where a given number of discrete sensor locations and target points are randomly placed. Each sensor has a sensing diameter of 25 m ($r_{s}=12.5$ m) covering a spherical space. We conduct two sets of experiments. In the first set of experiments, we fix both the number of sensor locations and the degree of coverage, and vary the number of target points; while in the second set of experiments, we fix both the number of sensor locations and the number of target points, and vary the degree of coverage. Note that each experiment is repeated 20 times with different random seeds and the average number of required sensors with a standard deviation is measured for comparison between different algorithms.

### 6.2. Performance Evaluation of DLDT

For the evaluation of an approximate algorithm with an approximation ratio, one important aspect is to examine how it actually performs within the proven performance bound in different scenarios. For that purpose, we compare GreedyDLDT with an optimal solution, which is obtained by using the GNU Linear Programming Kit (GLPK) package to solve DLDT under the ILP formulation. Also, for a more practical evaluation of the performance, we compare GreedyDLDT with a genetic algorithm (GA)-based heuristic approach for sensor deployment, which mainly follows the design and implementation of the genetic operators (i.e., crossover, translocation, and mutation) in [27] with the following adaptations:

- The surveillance region is extended from a 2D plane to a 3D space, and divided into a number of uniform contiguous voxels of unit size.
- The sensing model is changed from probabilistic sensing to Definite Range Law Approximation (Cookie Cutter) [28].
- The sensor type is changed from heterogeneous to homogeneous, and accordingly, no priority is given to any sensor during the population initialization.
- The cost function considers the number of target points that have been sufficiently covered and is penalized against the total number of target points multiplied by *k*, which is the required degree of coverage.
- The fitness function is redesigned to minimize the number of deployed sensors under the constraint imposed from the cost function.

**Figure 5:**
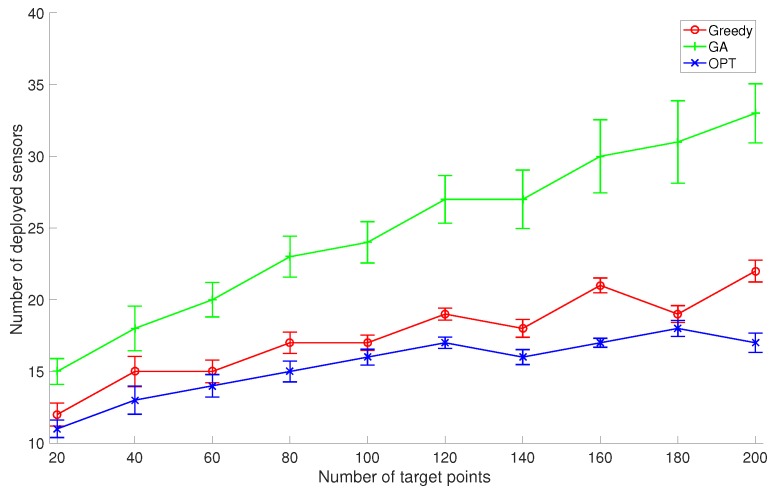
Comparison of the average performance with the standard deviation between GreedyDLDT (Greedy), Genetic Algorithm (GA), and Optimal Algorithm (OPT) for DLDT in response to a varying number of target points.

In the first set of experiments, we set the number of discrete sensor locations to 100 and set *k* to 2, and compare the average performance of GreedyDLDT with the GA-based approach and the optimal solution as the number of given target points varies from 20 to 200, as shown in Figure 5. The increasing trend in these performance curves indicates that more sensors need to be deployed to cover more target points. We observe that GreedyDLDT consistently outperforms the GA-based approach, and the number of deployed sensors computed by GreedyDLDT is close to that computed by the optimal solution. Also, GreedyDLDT exhibits a very stable performance as indicated by a small standard deviation in each problem size.

**Figure 6:**
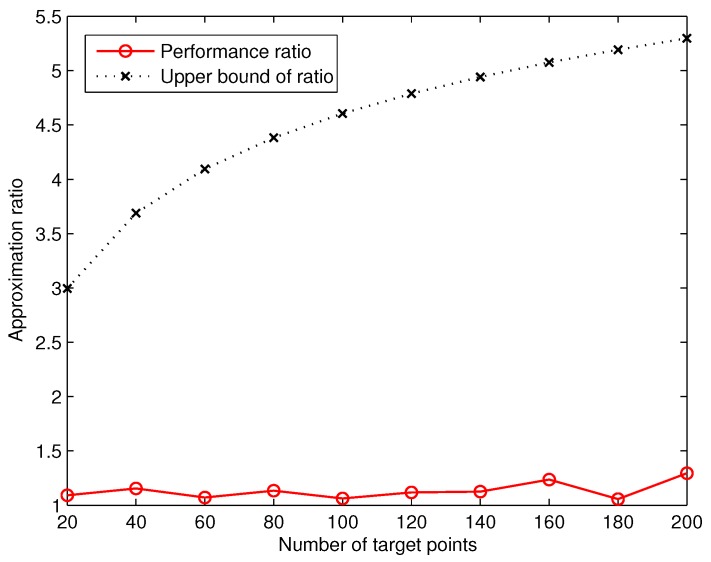
Measured average approximation ratio of GreedyDLDT for DLDT in response to a varying number of target points.

Based on the number of deployed sensors computed by GreedyDLDT and the optimal solution, we are able to measure the actual approximation ratio of GreedyDLDT. We plot in Figure 6 the measured average approximation ratio in comparison with the theoretical one. We observe that the actual approximation ratio of GreedyDLDT is much smaller than the upper bound (lnn+1) defined in Theorem 2, where *n* is the number of target points. These results shed light on the promising performance of the proposed method in solving such sensor deployment problems in practical applications.

**Figure 7:**
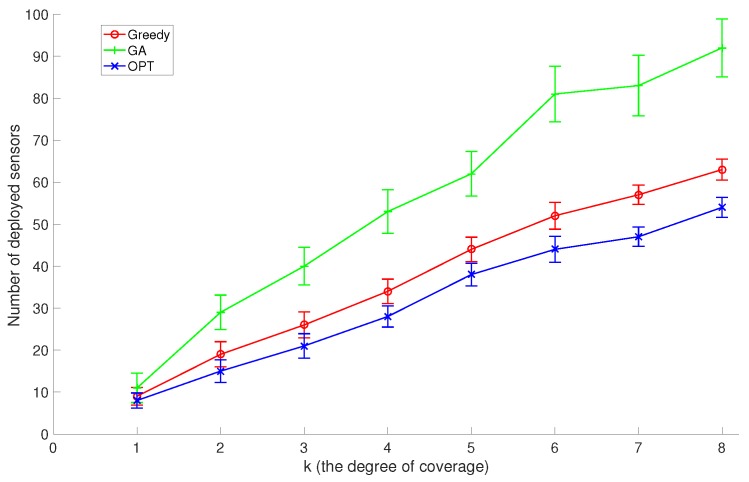
Comparison of the average performance with the standard deviation between GreedyDLDT (Greedy), Genetic Algorithm (GA), and Optimal Algorithm (OPT) for DLDT in response to varying *k* values.

In the second set of experiments, we set the number of discrete sensor locations to 200 and the number of target points to 100, and compare GreedyDLDT with the GA-based approach and the optimal solution with different *k* coverage values (the degree of coverage) varying from 1 to 8. As shown in Figure 7, the increasing trend in these performance curves indicates that more sensors need to be deployed to *k*-cover all target points as *k* increases. Again, we observe that GreedyDLDT consistently outperforms the GA-based approach, and exhibits a close-to-optimal performance with a high stability.

**Figure 8:**
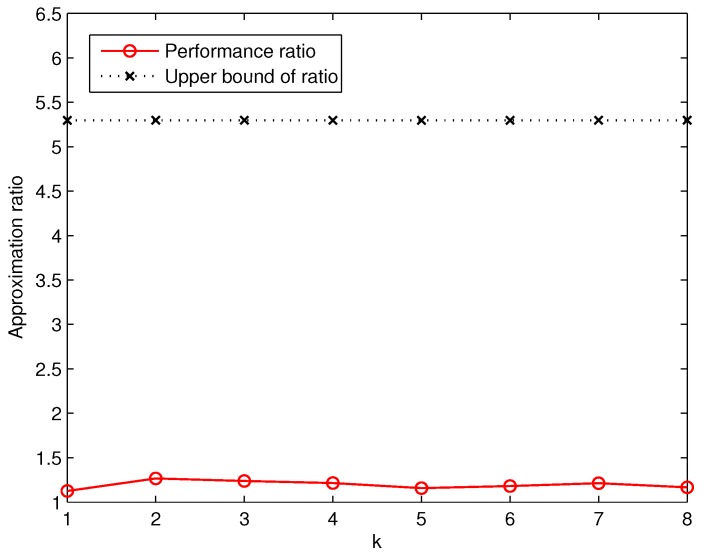
Measured average approximation ratio of GreedyDLDT for DLDT in response to varying *k* values.

Similarly, based on the number of deployed sensors computed by GreedyDLDT and the optimal solution, we are able to measure the actual approximation ratio in comparison with the theoretical one, as plotted in Figure 8. We observe that the actual approximation ratio of GreedyDLDT is no more than 1.3 in all the cases, while the upper bound in this set of experiments is about 5.4 in the worst case, which again strongly indicates the effectiveness of GreedyDLDT in practical use and its great potential for real-life deployment.

### 6.3. Performance Evaluation of GreedyDLDT in Practical Settings

In order to evaluate the performance of GreedyDLDT with realistic deployment constraints, we consider an exhibition hall at Zhejiang Provincial Museum, Hangzhou, China [29]. This two-story exhibition hall is roughly estimated to be 40 m × 60 m × 15 m, where surveillance sensors with a sensing distance of 5 m are constrained to certain locations with accessible mounting boards to achieve a 2-coverage for a varying number of exhibition items placed on exhibition stands scattered across the hall.

**Figure 9:**
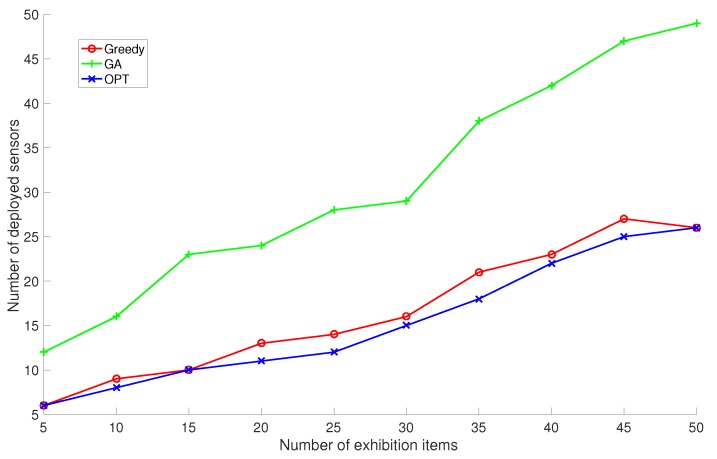
Performance comparison of GreedyDLDT (Greedy), Genetic Algorithm (GA), and Optimal Algorithm (OPT) for a museum exhibition hall with sensor mounting constraints in response to a varying number of exhibition items.

We run the proposed GreedyDLDT algorithm, the GA-based approach, and the optimal solution to determine the sensor deployment scheme in this exhibition hall as the number of exhibition items are increased from 5 to 50 at an interval of 5 items. The performance measurements in Figure 9 show that GreedyDLDT requires about only half of the sensors compared with GA to provide the same sensing quality and achieves a close-to-optimal performance in all the cases. Although the surveillance region in this exhibition hall is smaller than the cubic volume in the previous simulations and there are less target points (exhibition items) to be covered, we observe that even more sensors are actually required for coverage due to the particular constraints on sensor locations and the regular layout of exhibition stands.

### 6.4. Performance Evaluation of C-DLDT

**Figure 10:**
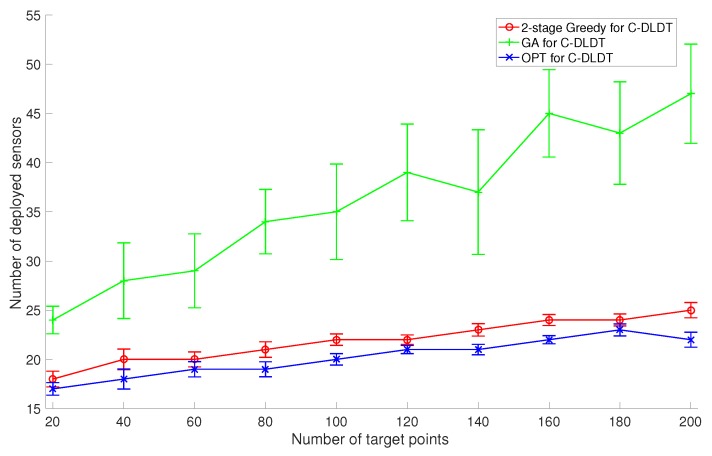
Comparison of the average performance with the standard deviation between the 2-stage Greedy Algorithm, Genetic Algorithm (GA), and the Optimal Algorithm (OPT) for C-DLDT in response to a varying number of target points.

**Figure 11:**
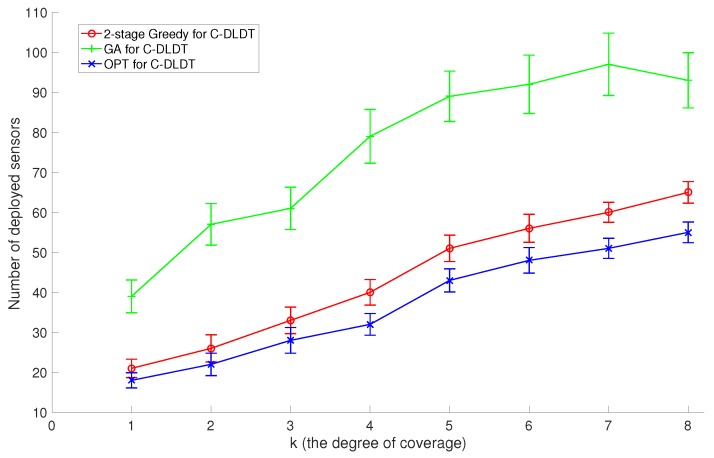
Comparison of the average performance with the standard deviation between the 2-stage Greedy Algorithm, Genetic Algorithm (GA), and the Optimal Algorithm (OPT) for C-DLDT in response to varying *k* values.

Similarly, we focus on the performance evaluation of C-DLDT because it serves as the base of the solutions to the connected version of the other three problems. We also run the proposed two-stage (first coverage and then connectivity) Greedy algorithm, the GA-based approach, and the optimal solution for C-DLDT to solve the two sets of problem instances created in Section 6.2. Note that in the GA-based approach, we incorporate connectivity (the percentage of connected sensors) as a penalty into the constraint. We plot the corresponding performance measurements in Figure 10 and Figure 11. We observe that the performances of these algorithms for C-DLDT are qualitatively similar to those for DLDT and C-DLDT requires more sensors to be deployed than DLDT, especially when there are only a limited number of sensors deployed for coverage. Furthermore, the performance curves for C-DLDT appear more flattened out than those for DLDT because it would require less extra sensors to be deployed for connectivity as the number of sensors that have been deployed for coverage increases.

## 7. Conclusions

We generalized and investigated a class of problems to deploy a minimum set of wireless sensors at candidate locations in constrained 3D space to achieve *k*-coverage of given target objects such that every point in the target objects is covered by at least *k* sensors. With different constraints on sensor locations and target objects, we formulated four sensor deployment problems: DLDT, DLCT, CLDT, and CLCT, which have been proved to be NP-complete.

We designed an approximate algorithm for DLDT with an approximation ratio of (lnn+1), and converted the other three problems to DLDT by using a discretization process. We proved that the number of divisions intersected by *m* circles in an infinite 2D area is tightly upper bounded by $\left(m^{2}-m+1\right)$, which is used to compute the approximation ratio of the algorithm for DLCT. We further considered the connected version of these constrained sensor deployment problems where all deployed sensors must form a connected network. An approximate algorithm was also designed for each of these connected deployment problems.

The simulation results show that the proposed deployment algorithms consistently outperform a genetic algorithm-based approach and achieve a close-to-optimal performance in small-scale problem instances. The measured approximation ratios in the simulations are generally much less than the theoretical upper bounds derived for the worst cases, which illustrates the efficacy of these algorithms in practical applications.
